# Recent developments in Friedreich’s ataxia: a state-of-the-art review

**DOI:** 10.1093/braincomms/fcag143

**Published:** 2026-04-20

**Authors:** Laura R Chapman, Heather Mortiboys, Pamela J Shaw

**Affiliations:** Sheffield Institute for Translational Neuroscience, School of Medicine and Population Health, University of Sheffield, 385a Glossop Road, Sheffield, S10 2HQ, UK; Paediatrics Unit, Sheffield Children’s Hospital, Clarkson Street , Sheffield, S10 2TH, UK; Sheffield Institute for Translational Neuroscience, School of Medicine and Population Health, University of Sheffield, 385a Glossop Road, Sheffield, S10 2HQ, UK; Sheffield Institute for Translational Neuroscience, School of Medicine and Population Health, University of Sheffield, 385a Glossop Road, Sheffield, S10 2HQ, UK; NIHR Sheffield Biomedical Research Centre, Sheffield Teaching Hospitals NHS Foundation Trust, Sheffield, S10 2JF, UK

**Keywords:** Friedreich’s ataxia, FRDA, mitochondria, Nrf2 activators

## Abstract

Friedreich’s Ataxia (FRDA) is a progressive disorder, which is autosomal recessive. It is one of the most common inherited ataxias in Europe, the Middle East, South Asia and North Africa. It is a life limiting condition, the time of onset until death is on average 36 years. A lack of certainty about specific phenotypic changes, has meant that, until recently, there have been no medications licensed for use in FRDA. Over the last 5 years, the development of omaveloxolone has stimulated increasing research into the mechanistic understanding of FRDA. We have conducted a critical review of all the studies published between 1999 and December 2024. The free text search terms were ((Friedreich’s Ataxia) OR (FRDA) OR (FRDA) OR (inherited ataxias)) AND ((recent developments) OR (new developments) OR (new treatments) OR (phenotype)). We identified common themes, for example cellular phenotypical changes, such as mitochondrial and iron dysregulation and common medications that have been trialled. We have analysed the relevant, available studies (*n* = 139), highlighting the underlying reasons for any reported discrepancies. The papers in this review demonstrate what we know about the frataxin deficiency, which causes mitochondrial dysfunction and iron dysregulation. We also discuss what is not known and how these gaps in knowledge can affect the development of new therapeutic approaches. We investigate the cellular phenotypical changes in FRDA that are either established or hypothesized in the current literature, review the medications that have been tested and their trial outcomes, and consider potential future approaches that could guide the development of new therapies for FRDA.

## Teaser

We review current advances in understanding FRDA and emerging therapies. We discuss the complex cellular and metabolic phenotype, involving the mitochondria and Nrf2 pathway. Although Nrf2 activators show promise for disease-modifying treatment, authors stress that precision medicine approaches and long-term clinical trials remain essential for development.

## Graphical Abstract

**Graphical Abstract:**
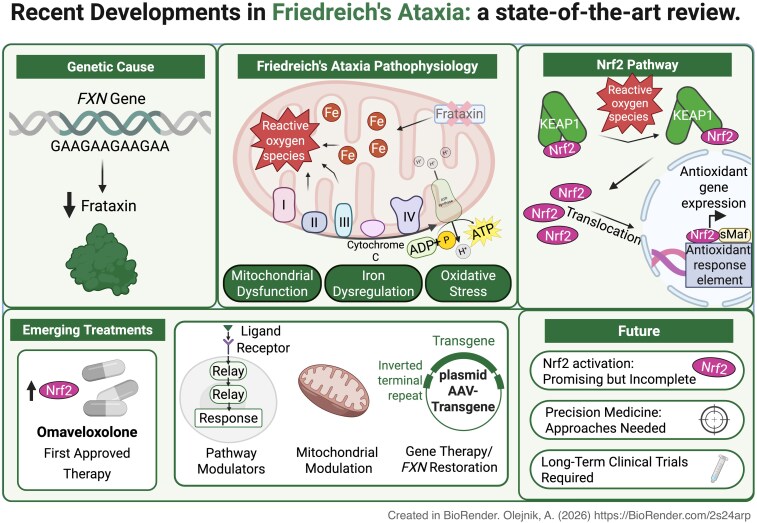

## Introduction

**Figure 1:**
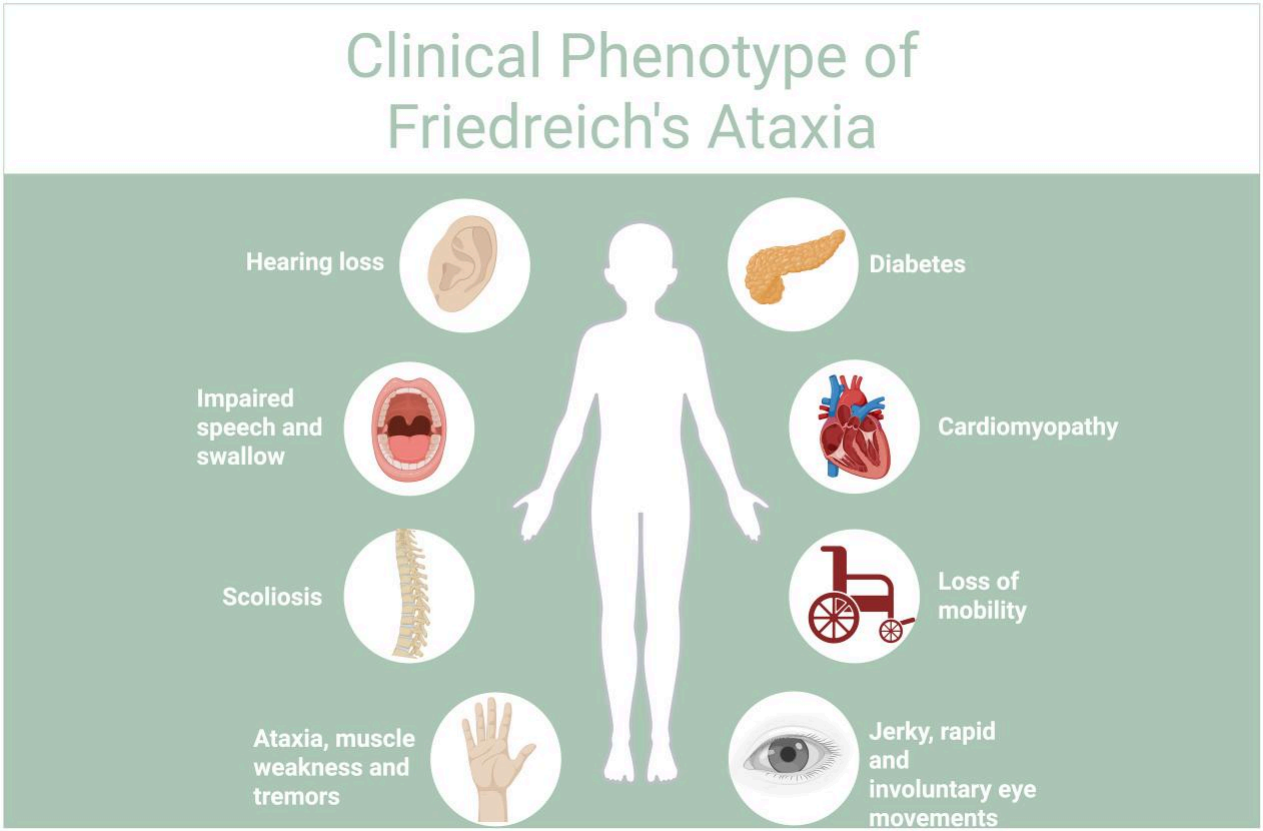
**Clinical phenotype in Friedreich’s ataxia.** The key clinical phenotypical changes seen in FRDA, including diabetes, cardiomyopathy, loss of mobility, jerky, rapid and involuntary eye movements, ataxia, muscle weakness and tremors, scoliosis, impaired speech and swallowing and hearing loss are highlighted. These clinical signs and symptoms vary depending on GAA repeat length and age of onset.^5-10^ Created in BioRender. Chapman, L. (2026) https://BioRender.com/fidttf8.

Friedreich’s Ataxia (FRDA) is one of the most common inherited ataxias in Europe, the Middle East, South Asia and North Africa.^1^ It is a progressive disorder, in which inheritance is autosomal recessive, hence males and females inherit equally. Its prevalence is 1 in 40,000, with an onset between 5 and 15 years of age. It is a life limiting condition, and the time of onset until death is on average 36 years.^2^ FRDA occurs at a global prevalence of 1 individual per every 22 000–50 000 people.^3,4^ The carrier frequency of pathogenic Frataxin (*FXN)* variants, typically a single pathogenic guanine–adenine–adenine (GAA) repeat expansion, is estimated at 1 in 60 to 1 in 100.^3,4^ The symptoms arise from progressive degeneration of the dorsal root ganglia (DRG), spinal cord pathways and cerebellar connections, resulting in proprioceptive sensory loss and progressive ataxia characterized by impaired coordination, balance difficulties and gait disturbance (Fig. 1). The clinical phenotype can vary significantly, creating a diagnostic challenge, with symptoms such as sleep disorders, dysphagia and neuropsychiatric symptoms present in some patients. It can also present with dysarthria, diabetes mellitus, scoliosis and hypertrophic cardiomyopathy.^5^ On average wheelchair use is required about 15 years after symptom onset. The commonest cause of death is cardiac due to hypertrophic cardiomyopathy and cardiac failure.^11^

FRDA is caused by the presence of a GAA-triplet expansion in the first intron of the *FXN* gene, causing a loss of function change in the frataxin protein. Healthy individuals typically have 5–30 GAA repeats, alleles with GAA repeats greater than 66 are considered pathogenic and FRDA patients may harbour 66 to 1700 repeats, commonly ∼600 to 1200.^8,12^ The most common defect is a homozygous GAA pathogenic triplet repeat found in ∼95% of FRDA patients, the other remaining 5% are compound heterozygous, with a GAA trinucleotide repeat expansion on one allele and a point mutation, deletion or insertion on the other.^5,10,13^ The larger the shorter allele GAA repeat, the more severe the disease is and the younger the age of onset.^6-10^ Frataxin is an important protein in iron homeostasis and general mitochondrial function. Therefore, a deficiency in this protein is considered to cause mitochondrial fragmentation, iron dysregulation, an increase in oxidative stress and loss of the mitochondrial respiratory chain function, leading to reduced production of ATP, dysregulation of cellular energy homeostasis and the progressive phenotype seen in FRDA.^14-24^

The specifics of these biochemical changes in FRDA and their relationship to physical symptoms are unknown, despite our ever-increasing understanding of the pathophysiology of FRDA. There are limitations in many of the recent studies, due to limited availability of patient-derived models, difficulties in reprogramming fibroblasts to relevant cell types (DRG neurons), the complexity of frataxin deficiency, patients’ variation in frataxin deficiency and clinical symptoms and low statistical power due to small sample sizes. There has been a recent focus on Nrf2 activators and mitochondrial dysfunction in neurodegenerative conditions, such as FRDA. With the new approval of omaveloxolone by the US Food and Drug Administration (FDA), there is a drive to develop more effective treatments for conditions such as FRDA. This present review aims to evaluate the recent research evidence emerging in relation to FRDA, to provide an overview of what we know about the clinical phenotypes of FRDA, the medications that have been trialled previously, outcomes and potential future treatment approaches, including genetic therapies. An outline of each drug that has been trialled is included in the Supplementary Materials.

## Materials and methods

### Search strategy and selection criteria

**Figure 2:**
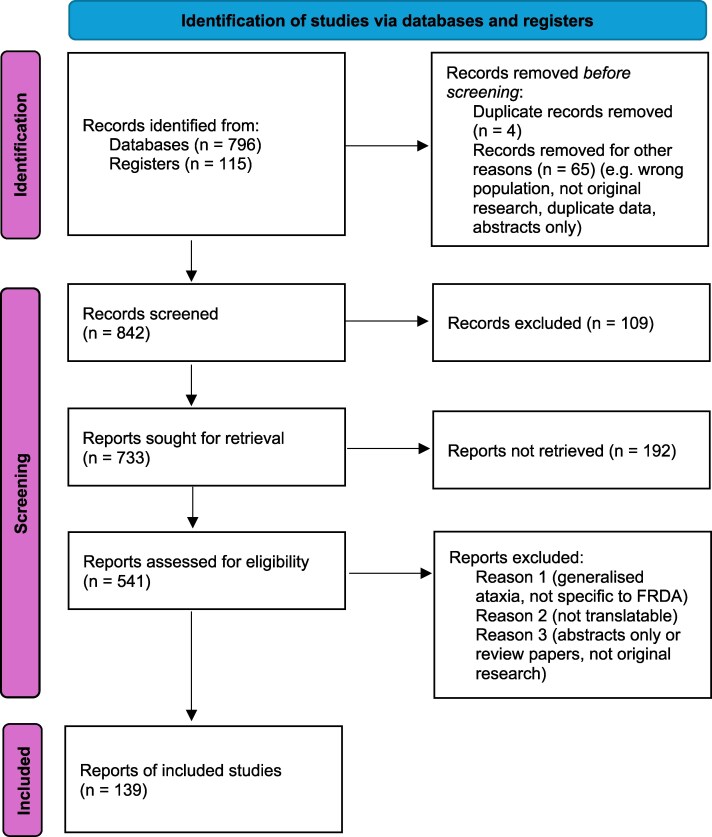
**Prisma chart.** A Prisma chart highlighting the number of studies involved in this critical review, how many databases were included and papers that were included and excluded.^25^.

The papers for this critical review were identified by searching PubMed Central and MedLine between 1999 and November 2024. The reference lists of these articles were searched to identify further relevant publications. The free text search terms were ‘recent’ [All Fields] AND ‘developments’[All Fields] AND (‘Friedreich ataxia’[MeSH Terms] OR (‘Friedreich’[All Fields] AND ‘ataxia’[All Fields]) OR ‘Friedreich ataxia’[All Fields] OR (‘Friedreich’s’[All Fields] AND ‘ataxia’[All Fields]) OR ‘Friedreich’s ataxia’[All Fields]). The inclusion criteria included papers published in English language or that could be translated. Only articles with publisher-provided English translations were included; no external translation services or software were used. The exclusion criteria were as follows: inability to access the full article text, papers which did not report clear outcomes, papers which could not be translated into the English language and papers not specific to FRDA. The final reference list was generated based on significance to the topics covered in this critical review. The PRISMA flow diagram is shown in Fig. 2.^25^

## Results

### Pathophysiology

*FXN* codes for frataxin, which is a mitochondrial matrix protein. It is essential for the assembly of iron–sulphur cluster (ISC) biogenesis, which is required for respiratory complex formation and frataxin deficiency that leads to mitochondrial respiratory defects and a reduction in mitochondrial membrane potential. In a healthy participant, it is normal to have <36 GAA repeats, in FRDA it is common for a patient to have ∼56–1300 repeats.^2^ The repeat expansion is unstable in meiosis and mitosis, meaning that it can increase or decrease in size when passed from mother to child. It is often seen to decrease in size when passed from a father to offsping.^2^ It is known to have post-zygotic instability, therefore increasing in size as people with FRDA get older.^2^ 95% of patients with FRDA are homozygous for GAA trinucleotide repeat expansions within intron one of the FRDA gene located on 9q13.1.^10^ The remaining 5% are heterozygous mutations, with a GAA trinucleotide repeat expansion and a point mutation. These differing point mutations can lead to variations in symptoms and make the condition harder to diagnose. It has been found that the size of the smaller GAA expansion (GAA1 allele) inversely correlates with age of onset and rate of disease progression.^6-10,26^

Frataxin is highly expressed in the DRG, spinal cord and cerebellar dentate nuclei. Hence, the main sites of disease in the nervous system are the DRG, dentate nuclei of cerebellum, posterior columns, spinocerebellar tracts and corticospinal tracts of the spinal cord and peripheral nerves.^2^ The pathophysiology of FRDA is due to complex biology of the effect of reduced frataxin on mitochondrial function, iron dysregulation, impaired Nrf2 signalling, oxidative stress and multi-organ damage. The interactions and these effects are what cause the phenotypical changes seen in FRDA.^14-24^ These are discussed in more detail in the following sections.

### Pathophysiology—frataxin deficiency

FRDA is caused by a deficiency in the frataxin protein. *FXN* is located on 9q21.11,and encodes a mitochondrial protein, which regulates mitochondrial iron transport and respiration. In FRDA, frataxin levels are 5–35% of the normal level seen in healthy individuals.^14,27^ A study focusing on inducible siRNA investigated the specific consequences of frataxin deficiency and the order in which they occur.^15^ These authors reported that the first change caused by a deficiency in frataxin is a defect in cytoplasmic iron–sulphur proteins, leading to oxidative damage, stress and activation of the unfolded protein response associated with neurological disease. The secondary changes found were due to protein oxidative damage, copper-zinc-superoxide dismutase (SOD1) was induced, as was the unfolded protein response, then mitochondrial aconitase activity declined and there was a decrease in haem-containing cytochrome c protein, and transcriptional induction of the haem-dependent transcripts ALAS1 and MAOA.^15^

The mechanism of the loss of *FXN* expression has been hypothesized to be caused via multiple different effects, including the induction of the GAA repeat-induced DNA triplex structures, also known as ‘sticky DNA’, disrupting *FXN* gene transcription^28-30^ the formation of R-loop structures acting as triggers to promote *FXN* and FMR1 silencing, preventing transcription^31^ repressive heterochromatic structures extending to the *FXN* promoter, causing a reduction in initiation of the transcriptional process^32,33^; this is exploited by histone deacetylase (HDAC) inhibitors, which do not fully reverse the gene silencing effects.^34,35^ These changes in frataxin levels lead to mitochondrial dysfunction, which causes the phenotypical changes seen in FRDA.

### Pathophysiology—mitochondria

There is increasing evidence that neurodegenerative diseases stem initially from neuronal dysfunction rather than cell death itself.^17^ Mitochondria are especially important in neurons, aiding with neuronal development and maintenance, specifically energy production, calcium homeostasis, maintenance of membrane potential, dendritic transport and the release and reuse of neurotransmitters at synapses. Therefore, it is unsurprising that dysfunction of mitochondria play an important role in many disease processes. Mitochondrial dysfunction in FRDA is not fully understood. However, there is evidence that iron accumulation, oxidative stress, Fe––S cluster deficits, ATP depletion, mitochondrial fragmentation and impaired mitophagy are all likely to contribute to progressive neurodegeneration and cardiomyopathy.^14-24,36^

A key function in healthy mitochondria is the production of energy in the form of ATP through oxidative phosphorylation, via the electron transport chain (ETC). In the presence of frataxin deficiency, as seen in FRDA, impaired oxidative phosphorylation and ATP production are seen.^19^ The ETC consists of five main protein complexes that drive ATP production, including complex I (NADH:ubiquinone oxidoreductase), complex II (succinate dehydrogenase), complex III (cytochrome bc1 complex), complex IV (cytochrome c oxidase) and complex V (ATP synthase). A report describing the Yfh1p protein in yeast (yeast homolog of frataxin) found that it physically interacts with succinate dehydrogenase, specifically in Sdh1p and Sdh2p, which are part of the complex II of the mitochondrial ETC.^21^ These findings were extended to humans and demonstrated that human frataxin also interacted with these subunits, suggesting that frataxin plays a direct role in complex II function in human mitochondria.^21^ This was also seen in frh-1 worms (the worm homolog of frataxin), highlighting that lower levels of frataxin increase oxidative stress, reduce the lifespan and cause lethality in a mitochondrial complex II mutant. Thus, mitochondrial respiratory chain defects are likely involved in the pathophysiology of FRDA.^22^ Mouse models have also shown a significant reduction of complex I and II enzyme activities in frataxin knock-in/knockout models, when compared to age-matched controls.^37^ Frataxin deficiency, therefore, leads to mitochondrial respiratory chain defects, reduced ATP production and decreased energy supply to high-demand tissues, such as neurons and cardiomyocytes. However, further research needs to be done in FRDA patient cell lines and different cell types, to investigate in more detail whether this is a decrease in activity or dysregulation of protein complexes and the specific mechanisms of these defects. These respiratory chain complexes could be studied further as potential therapeutic targets.

The importance of the organization of the mitochondrial network and its relationship to energy production has become increasingly apparent. Human fibroblasts with genetic changes in the subunits of the respiratory chain complexes have been shown to also have a more fragmented mitochondrial network.^23^ In FRDA, the increase in oxidative stress in the mitochondria leads to an increase in mitochondrial fragmentation and impaired mitophagy in frataxin-deficient cells. This has been shown in multiple yeast models lacking frataxin, indicating that an increase in oxidative stress induces fragmentation of the mitochondria, which is directly implicated in the reduction of cellular ATP levels.^24,38^ The mitochondrial changes are not only limited to morphology and the ETC, but iron dysregulation is also seen in the presence of frataxin deficiency. A more extensive assessment of other important mitochondrial functions is currently lacking.

### Pathophysiology—iron accumulation

Frataxin is a mitochondrial protein that is essential for iron homeostasis. A lack of frataxin in FRDA leads to a defect in iron homeostasis, via downregulation of mitochondrial ISC synthesis, changes to iron storage, haem synthesis and changes in expression of molecules involved in cellular and mitochondrial iron uptake.^39,40^ The resulting excess iron accumulation causes an increase in reactive oxygen species, increasing oxidative stress and mitochondrial dysfunction.

Iron is required for brain development and normal neurological function and is essential for the development of synapses, neurotransmitter turnover and cellular respiration.^41^ It is a potent oxidant and can promote cellular damage; hence, precise homeostasis is essential. The mechanisms leading to mitochondrial iron dysregulation and iron accumulation in FRDA are complex. Studies have shown that a lack of frataxin in human or mouse caused defects in ISC-dependent enzymes. These defects were seen prior to detectable mitochondrial iron accumulation, but have subsequently led to this accumulation.^36,42^ It is known that there is regional iron accumulation in the DRG, cerebellar dentate nucleus and the heart and generalized ISC enzyme deficits in FRDA.^43,44^ ISC components are highly adaptable enzyme co-factors, that are essential for the function of the mitochondria, nucleus and cytoplasm.^18^ They facilitate catalysis of chemical reactions and are vital as electron transfer devices. Most species have ISCs and depend on them for viability.^18^ Mitochondrial ISC synthesis is key to the maintenance of numerous vital enzymatic activities, as well as maintenance of cellular iron homeostasis. Therefore, partial defects in the mitochondrial ISC can cause severe detrimental effects, including impaired energy metabolism, oxidative damage and loss of mitochondrial DNA integrity. This has also been extensively studied in *Saccharomyces cerevisiae* mutants, in which complete loss of ISCs is not compatible with life.^45-47^ When these are reduced, the cell responds with large cellular uptake of iron, increasing the iron content within the mitochondria. This is one of the mechanisms by which iron is dysregulated in FRDA.^15,44,48-50^ In addition to this, frataxin when operating properly as a functioning protein also helps iron regulation.^51^ It binds iron, donates iron to other iron-binding proteins, oligomerises it, stores iron and controls iron and redox chemistry. Therefore, the presence of a defect in the function of frataxin and a reduction in mitochondrial ISC can cause progressive accumulation of oxidative damage.^51^

The increasing understanding of the exact mechanism of iron dysregulation allows for therapeutic approaches, such as iron chelators. A multicentre, double-blinded, placebo-controlled trial of an iron chelator, deferiprone, reported that after 6 months of treatment, there was a reduction in excess deposited iron in the brain, reduced neuropathy and ataxia, with limited side effects. This trial also demonstrated radiological improvement, with reduction of iron load in the basal ganglia and a trend to slowing of disease progression.^52,53^ This highlights the importance of understanding the pathophysiology of the disease in detail, to allow development of disease-modifying treatments. The different treatments that have been trialled for FRDA are discussed below in more detail.

The iron dysregulation is thought to also contribute to cardiomyopathy, the accumulation of iron in the myocardial cells, leading to increased oxidative damage in the heart. A previous mouse model used a gene-targeting approach to generate frataxin deficiency in the heart, which caused cardiac hypertrophy. These models highlighted that intramitochondrial iron accumulation occurs at the onset of the pathology, after the activation of Fe-S-dependent enzymes, leading to the cardiac hypertrophy seen in FRDA.^36^

### Pathophysiology—cardiomyopathy

Cardiomyopathy is the most common cause of death in FRDA, due to a progressive hypertrophic cardiomyopathy, which progresses to heart failure and death. Despite this, there has not been much conclusive research into the mechanism behind this. The cardiac phenotype in FRDA is quite variable, usually presenting around the second to fifth decade of life.^54^ However, it is commonly characterized by increased symmetrical, concentric thickening of the ventricular wall, with a normal left ventricular (LV) cavity and normal systolic function.^55^

It has previously been found that the severity of the LV hypertrophy is related to the number of GAA repeats.^55^ This highlights the importance of frataxin in the function of a healthy heart and specifically in the modulation of cardiac hypertrophy. This report is a relatively small study, with only 44 FRDA patients; however, this increasing severity, with increasing GAA repeats, has been seen with other features of FRDA, for example increased mitochondrial dysfunction.^6-10,26^ Despite this study being from 1997, the exact mechanism of the frataxin deficiency leading to cardiomyopathy is unknown. A more recent study of 25 patients with FRDA compared with healthy controls found that LV mass was most pronounced in patients with a larger GAA repeat size (GAA1 repeats >600), earlier age of onset (<16 years) and shorter duration of disease (<15 years).^56^ It was also suggested in this study that cardiac thinning occurred with prolonged disease, due to LV mass decreasing with longer disease duration (>15 years), independently of GAA repeat size.^56^ This highlights the importance of the extent of the frataxin deficiency, the variation within FRDA patients depending on this and documents how the phenotype can change over time.

It is thought that the cardiac changes are due to deficient mitochondria in the heart, which are unable to provide sufficient energy to the muscle.^57^ Interestingly, women with FRDA demonstrate larger LV ejection fractions than men on echocardiographic studies, potentially decreasing the likelihood that women will compensate for the cardiac insufficiency seen in FRDA with ventricular hypertrophy.^2^ This highlights further variation within the FRDA phenotype, and the many factors involved in the complex phenotype, increasing the difficulties for unravelling the underlying the pathophysiology.

### Pathophysiology—Nrf2

**Figure 3:**
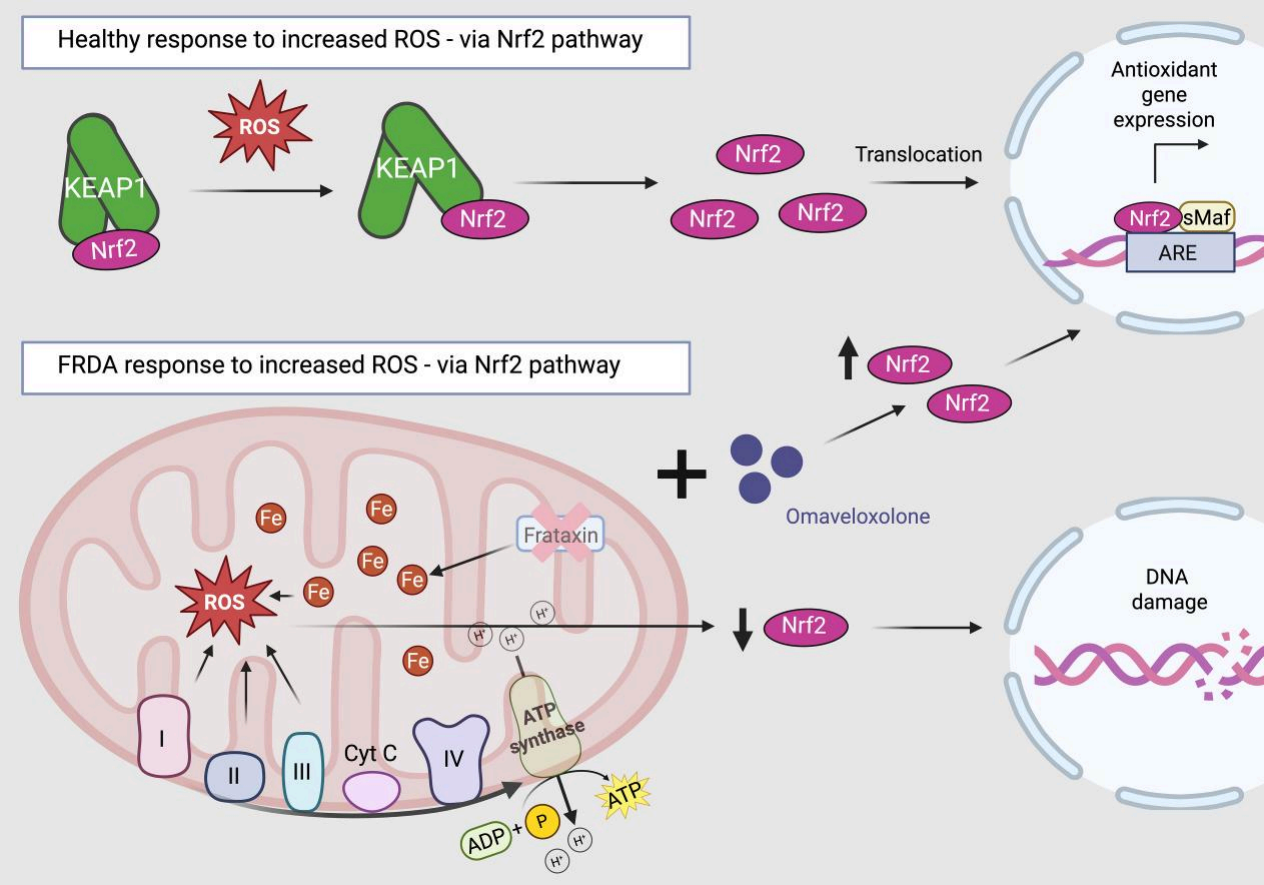
**The Nrf2 pathway and the oxidative stress response.** In response to reactive oxygen species (ROS), the Nrf2 pathway is activated when Nrf2 dissociates from KEAP1, causing translocation to the nucleus, and promotes the transcription of antioxidant and cytoprotective genes to mitigate oxidative stress. In Friedreich’s ataxia, reduced levels of Nrf2 impair this protective pathway. Coupled with increased ROS—secondary to frataxin deficiency and elevated free iron—this results in heightened oxidative and DNA damage and a diminished cellular antioxidant response. Omaveloxolone is a Nrf2 activator, which disrupts KEAP1-Nrf2 binding, preventing KEAP-1 mediated degradation of Nrf2 and allowing Nrf2 to accumulate in the nucleus. This increases the pool of free Nrf2 that can enter the nucleus and drive transcription of antioxidant and cytoprotective genes, boosting the cell’s antioxidant capacity. Created in BioRender. Chapman, L. (2026) https://BioRender.com/kylzvm0.

Another prominent feature in the complex pathobiology of FRDA is the transcription factor nuclear factor erythroid 2 related factor 2 (Nrf2). There has been a lot of recent interest in the role of this transcription factor in neurodegenerative disorders. Nrf2 controls the activity of over 250 genes by binding to antioxidant response elements in their promoter regions.^58,59^ Nrf2 target genes have been shown in microarray analyses of blood samples from FRDA patients to be downregulated and an impairment has been seen in the Nrf2 signalling pathway in cultured fibroblasts from FRDA patients.^60-62^ NRF-2-regulated genes have roles in antioxidant and anti-inflammatory actions, electrophile detoxication, cell metabolism, proliferation and differentiation, and general cytoprotection.^63^ There are many downstream targets of this biological pathway, including nicotinamide adenine dinucleotide phosphate (NAD(P)H), NAD(P)H quinone dehydrogenase 1(NQO1), heme oxygenase 1 (HMOX1), glutamate-cysteine ligase (GCLC) and glutathione S-transferase. Therefore, Nrf2 has an important role and functions as a key regulatory switch, moving between the cytoplasm and the nucleus. Under normal conditions, Nrf2 levels remain low in the cytoplasm due to continuous degradation via the ubiquitin-proteasome system, a process mediated by Kelch-like ECH-associated protein-1 (KEAP1). KEAP1 is key to this pathway, as it is rich in cysteine residues (25 in mice, 27 in humans) and binds Nrf2 in the cytoplasm positioning it for continuous ubiquitination and degradation.^64^ These highly reactive cysteine residues allow compounds to induce conformational change that disrupts Nrf2 ubiquitination, activating Nrf2. Nrf2 binds KEAP1 via ETGE and DLG motifs in a ‘two-site substrate recognition’ mechanism. KEAP1 also interacts with the CUL3-based E3 ligase complex to mediate Nrf2 polyubiquitination. Under oxidative stress or compound binding, KEAP1 cysteines undergo conformational changes, weakening the DLG interaction while ETGE remains bound, disrupting Nrf2 inhibition.^64-68^ KEAP1 targets Nrf2 for degradation by binding to it, but under oxidative or electrophilic stress, specific cysteine residues in KEAP1 become oxidized, disrupting this interaction. This allows Nrf2 to accumulate and translocate to the nucleus, where it activates the transcription of genes involved in antioxidant defence and cellular protection.^58,59^ In FRDA, it is thought that impaired Nrf2 activation may contribute to disease progression, leading to inadequate cellular defence against oxidative damage (Fig. 3).

In various FRDA cell models—including DRG and Schwann cells from the YG8R mouse model DRG, fibroblasts derived from FDRA patients and neuroblastoma-derived cell lines with frataxin knockdown via shRNA—reduced expression of Nrf2 has been consistently observed compared to controls.^61,69-71^ These studies also demonstrate that FRDA cells exhibit heighted sensitivity to oxidative stress. Upon induction with oligomycin (an ATPase inhibitor), SOD1 levels were moderately increased, while glutathione (GSH) levels were significantly decreased, changes directly linked to diminished Nrf2 expression.^61^ Furthermore, in control cells, Nrf2 was found to be bound to phalloidin-reactive actin stress fibres via KEAP1.^61^ In contrast, in FRDA cell lines, Nrf2 lacked this tethering to a filamentous structure and was predominantly located at the cell periphery.^61,71^ This mislocalization impairs Nrf2’s nuclear translation in FRDA cells in comparison to controls, and reduces expression of target genes, such as SOD1, NQ01.^61,69-71^ Notably, frataxin expression has been shown to be significantly correlate with Nrf2 expression.^69^

Overall, disruption of the Nrf2 signalling pathway in FRDA leads to decreased antioxidant enzyme production, defective ISC causing mitochondrial iron dysmetabolism. These factors collectively increase hydrogen peroxide production, contributing to the hypersensitivity to oxidative stress seen in FRDA cells. These findings support the use of Nrf2 activators as potential treatments in FRDA to enhance the antioxidant response and mitigate the neurodegeneration that occurs in FRDA.^61^

### Clinical trials and potential disease-modifying medications for FRDA

The lack of understanding and of a clear biochemical biomarker has hindered FRDA clinical trials. The most common method of assessing trial outcomes is by using the modified Friedreich’s Ataxia Rating Scale (mFARS), an internationally recognized scale. However, there are limitations of this scale: there is a degree of subjectivity from the examiner assessing the patient, and these are discussed in detail in recent reviews.^72-75^ The lack of a biological biomarker of disease means that currently clinical symptoms are the best way to assess outcomes in therapeutic trials.^76,77^ Other challenges in developing medications for FRDA include identifying medications with strong CNS penetration that specifically target the DRG neurons affected in FRDA whilst minimizing off-target effects and adverse side effects. In this part of the review, previous and ongoing trials of different therapeutic options that can potentially help improve the disease course of FRDA are discussed.

### Nrf2 activators—omaveloxolone

A drug recently approved as the first disease-modifying treatment for FRDA by the FDA is omaveloxolone. This is an Nrf2 activator. It prevents Nrf2 degradation by binding to KEAP1, leading to an improved function of complex 1 in the electron transfer chain and restoring mitochondrial dysfunction.^78^ In February 2023, the FDA has approved the use of omaveloxolone for FRDA patients aged 16 years and older. This decision was supported by the efficacy and safety data from MOXIe Part 2 trial and a post hoc propensity-matched analysis of the open-label MOXIe Extension trial.^79^

Omaveloxolone has been shown to protect the maintenance of normal mitochondrial membrane potential in response to oxidative challenge and prevent hydrogen peroxide-induced oxidative cell death in fibroblasts obtained from patients with FRDA.^80^ The Nrf2 pathway is also involved in the cellular response to oxidative stress and activation of this pathway is neuroprotective in model systems. It has also been shown in studies to aid with recovery in ejection fraction, stroke volume and cardiac output.^80^

The phase II MOXIe trial involved 103 patients randomized to drug or placebo.^80,81^ Patients were given 150 mg of omaveloxolone daily—the trial end point was a change in mFARS at week 48. They found that the group taking omaveloxolone had significantly lower mFARS score (*P* = 0.014) (− 2.40 ± 0.96; lower scores are associated with lesser disability) in the full analysis set population (NCT02255435). The common side effects reported were an increase in liver enzymes (alanine transaminase and aspartate aminotransferase), headache, nausea, abdominal pain, fatigue, diarrhoea and musculoskeletal pain.^80,81^ Therefore, with careful monitoring of liver function tests, it was deemed safe and with significant benefits of shown by the lower mFARS score.

The aim with medication is to improve disease-related symptoms and slow progression of disease. It is therefore important to allow children access to these medications. There is an ongoing clinical trial at the Children’s Hospital of Philadelphia. It is an open-label trial, phase 1, study (BOLD) to evaluate the safety and efficiency of omaveloxolone in participants ≥2 to <16 years of age with FRDA (NCT06054893).^78^ This study is focusing on the safety of the medication in this age group, how the pharmacodynamics apply in children and any changes during puberty. The study will be a large-scale longitudinal study, allowing for a large follow-up period of up to 240 weeks, to allow long-term evaluation of the trial data. These changes and treatments being developed for FRDA highlight the importance of the Nrf2 pathway and will pave the way for further research into compounds such as KEAP1 inhibitors and other Nrf2 activators.

### Antioxidants

Antioxidants have been considered beneficial in FRDA. It is postulated that decreased mitochondrial respiratory chain function, iron accumulation and subsequently increased oxidative stress are part of the disease process, hence the potential benefit of antioxidants. Therefore, it is not surprising that an American study found that 64% of participants with FRDA took at least one antioxidant.^82^ These commonly included coenzyme Q10 (CoQ_10_),, vitamin E, idebenone, selenium or N-Acetylcysteine (NAC). They had limited adverse events, with one patient reporting easy bruising and one patient reporting nausea when used in conjunction with NAC/selenium. These data highlight the minimal side effect profile. Vitamin E was the most commonly taken anti-oxidant. This makes it difficult to assess their effectiveness, as many trial participants have stated they believe them to be useful and would not want to halt the use of these to participate in a clinical trial of other potential therapies.

In the UK, CoQ_10,_ also known as ubiquinone, is often given to patients when diagnosed with FRDA, due to its potential benefits, good tolerability and low side effect profile.^83^ It is a lipophilic substituted benzoquinone, which is endogenously synthesized in every cell. It is specifically part of the respiratory chain in the inner mitochondrial membrane, acting as a mobile electron and proton transporter from complex I (NADH: ubiquinone reductase) and complex II (succinate: ubiquinone reductase) to complex III (ubiquinone cytochrome *c* oxidase) in the inner mitochondrial membrane. Given the evidence that the respiratory chain is affected in FRDA, there is a clear rationale for administering CoQ_10_.^84^

In an open-label study of 10 FRDA patients, participants were given 400 mg CoQ_10_ plus 2, and 100 IU vitamin E per day. However, they showed no consistent neurological benefits after 6 months, despite the CoQ_10_ levels increasing and cardiac and skeletal muscle bioenergetic profile improving after 3 months.^85^ In a further 4-year follow-up study involving 77 patients, it was found that seven showed some neurological improvement, with significant and persistent improvement in cardiac and skeletal muscle bioenergetics.^86^ The trial reported varying results, with changes in total international cooperative ataxia rating scale (ICARS) and kinetic scores better than predicted for these seven patients, but the posture, gait and hand dexterity scores progressed as predicted, i.e. showed no improvement. In all patients the echocardiogram results showed a significant fractional shortening at 35 and 47 months. When a higher dose of 600 mg CoQ_10_ plus 2100 IU vitamin E per day was assessed, it showed no additional benefit compared to the lower dose.^87^ This double-blinded study also found that at baseline, serum CoQ_10_ and vitamin E levels were significantly decreased in FRDA patients in comparison to controls. However, despite significantly increasing the levels of CoQ_10_ through this study, the ICARS did not change significantly.

A derivative of CoQ10 which provides increased absorption and tissue distribution, is idebenone.^83^ It is an antioxidant medication that increases the production of adenosine triphosphate via facilitating the flux of electrons along the mitochondrial ETC.^88^ It was seen in multiple trials to improve the ataxia rating scales; however, the trial results were inconsistent. It was initially approved in the USA and Canada, however later withdrawn from clinical trials once found to be not very effective. A large-scale randomized control trial in the US of 48 patients aged 9–17 years found there was no significant dose-related change or when compared to placebo over 6 months in urinary 8-hydroxy-2'-deoxyguanosine (8OH2'dG) (a marker of oxidative DNA damage), ICARS, the FRDA rating scale (FARS) or in a survey of activities of daily living.^89^ These results were also echoed in the phase 3, double-blind, placebo-controlled trial of 70 8–18-year-old ambulatory patients, in which they found idebenone did not significantly alter neurological function after 6 months.^90^ The differing disability level of patients with FRDA and the subjectivity of rating scores, such as FARS, can cause a large variation in trial outcomes. There is a large variation in severity and phenotype of patients, many trial criteria only include ambulatory patients, which is not representative of a large cohort of FRDA patients, therefore these medications may not be effective in all FRDA patients.

These inconsistent results contributed to the withdrawal of the product. One prospective open label trial looking at 38 patients stated that there was a significant reduction in LV mass of more than 20% in half of the patients after 6 months of treatment.^91^ However, another randomized, double-blind, controlled study, looking at 70 paediatric patients, found that idebenone did not decrease LV hypertrophy or improve cardiac function,^92^ therefore demonstrating no benefit from the medication. These inconsistencies are what makes the research difficult in evaluating the efficacy of new medications. Factors potentially contributing to these inconsistencies include a large variation in response in patients, differing clinical phenotypes, misunderstanding of the phenotype, variation in the disease stage, small sample sizes or subjectivity in rating scales.

Another antioxidant that has previously been trialled is resveratrol (35,4′-trihydroxy-trans-stilbene) which is a polyphenol. It is a phytoalexin that acts against pathogens, including bacteria and fungi. As a natural food ingredient, numerous studies have demonstrated that resveratrol possesses a very high antioxidant potential.^93^ Despite its potential benefits, it has very poor solubility, bioavailability and many adverse effects. In previous studies, it has been shown to have a neuroprotective effect, including improving neurological function and biogenesis through the SIRT1/AMPK/PGC1a pathways, preventing the deleterious effects triggered by oxidative stress in other neurodegenerative diseases including Alzheimer’s, Huntington’s and Parkinson’s diseases.^94-96^ An open-label, non-randomized trial in FRDA patients, looking at two different doses, found a significant improvement in neurological function at higher doses, but not at lower doses.^97^ There were also significant improvements in audiologic and speech measures and in oxidative stress marker plasma F2-isoprostane in the high-dose group. No serious adverse events were recorded in this trial. This study is limited due to having a very low sample size of only 12 patients per dose, which reduces the statistical power of the study. It also had a short follow-up period and the variability of severity and progression in FRDA patients is so prominent, that including only12 participants is not enough to determine whether a medication was successful or not. Nonetheless, given their low side effect profile and potential for benefit, the use of antioxidants may be justified, as the potential advantages outweigh the minimal risks.

### Iron chelation

The iron chelator deferiprone has been investigated as a potential treatment for FRDA. In FRDA, excess iron accumulation occurs in the mitochondria, due to frataxin deficiency, leading to impaired iron metabolism. The removal of this excess iron could have the potential to slow disease progression and improve neurological symptoms. Deferiprone has been shown to be effective in cell and animal models of FRDA, and a 6-month randomized control trial of five FRDA patients showed that it reduced disease progression and cardiac hypertrophy.^98^ These results are limited due to the low sample size, reducing the impact of the report. The patients were also given idebenone, so the results may reflect the combination of the two medications, or the impact of just one of them.

Deferiprone may have a dose-dependent benefit. A randomized, double-blinded, placebo-controlled study found that 20 mg/kg/day of deferiprone had a benefit in patients with less severe disease.^99^ It led to an improvement in ICARS, FARS and kinetic function. This was not seen when all doses were assessed, therefore reducing the power of this outcome, due to a smaller sample size. They found that a 20 mg/kg/day or 40 mg/kg/day dose both reduced the LV mass index, however the 40 mg/kg/day dose appeared to worsen the FARS and ICARS score.

To ensure the iron chelation targets the parts of the brain affected in FRDA, it is important to undertake brain imaging. A report by Boddaert *et al*.^50^ assessed the possibility of reducing brain iron accumulation in FRDA patients. Brain MRI scans in FRDA patients compared to aged-matched controls highlighted smaller and irregularly shaped dentate nuclei, with significantly higher H-relaxation rates R2* indicating regional iron accumulation. They found that a 6-month treatment with deferiprone significantly reduced the store of toxic iron in the brain using MRI T2* following L1 treatment. These benefits were also seen clinically, coinciding with a reduction in ataxic gait and neuropathy. Therefore, there is a potential benefit from the use of deferiprone and further research into the exact pathophysiology of the increased iron accumulation and targets for this is required.

### Erythropoietin treatment

The use of erythropoietin (EPO) has been studied in multiple reports as a potential therapeutic agent for FDRA due to its effect on iron metabolism and potential effect to increase frataxin levels *in vitro* and *in vivo*.^100-105^ EPO is currently used to treat severe anaemia and is known to decrease apoptotic cell death of cardiac myocytes, by recruiting endothelial progenitor cells, enhancing angiogenesis and neovascularisation. It is also thought to reduce the levels of interleukins and other cytokines, hence acting as an anti-inflammatory agent.^105^ Therefore, EPO may be beneficial in FRDA due to its potential to increase frataxin levels, reduce oxidative stress and improve overall mitochondrial function.

A large multicentre, double-blind, phase II trial was conducted of EPO in FRDA.^100^ This study specifically looked at a carbamylated EPO, Lu AA24493 (CEPO). The duration of symptoms and time to diagnosis ranged in both placebo and EPO groups, there was a spread of ∼3 years and ∼10 years, respectively. This study was limited as it did not include non-ambulatory patients, and any patients with a longer duration of FRDA, which may affect the therapeutic potential of EPO in a chronic disease. EPO was seen to be safe; it is used currently in other conditions and there were no adverse events that led to discontinuation of the study, the overall adverse events were similar in both groups. However, no significant effects on frataxin levels or functional rating scales were observed.

These results differ from other studies, which found that EPO significantly increased frataxin levels, for example, see Boesch *et al*.^101^, who conducted a 6-month open-label clinical pilot study of eight adults and one dose of EPO (2000 units IU three times weekly, via subcutaneous injection). Boesch *et al*. found that FARS and SARA significantly improved, frataxin significantly increased and markers of oxidative stress (urinary 8-OHdG and peroxide) decreased.^101^ The other important factor when validating a drug is its safety profile. Four participants out of eight had an increase in haematocrit and required phlebotomies. Therefore, there would have to be strict parameters and monitoring in place, and this may not be feasible for patients or safe for longer term use. A similar sized open-label clinical pilot study, including 12 patients looked at taking 5000 units of recombinant human EPO three times a week, showed promising outcomes.^105^ It investigated *FXN* levels measured in isolated lymphocytes by enzyme-linked immunosorbent assay, urinary 8-hydroxydeoxyuanosine and serum peroxides. They also found that there was a persistent and significant increase in *FXN* levels after 8 weeks and a reduction in markers of oxidative stress. These data would need to be replicated on a larger scale and over a longer time period. Another study looking at dose response to EPO found that frataxin significantly increased over 3 months; however, SARA scores did not change.^104^ This study would need to be conducted over a longer period of time to see if there is a clinical impact for patients, as despite an increase in frataxin, if this does not alter or halt symptom progression, patients may not be willing to take this medication. The NADH/NAD ratio, an indicator of mitochondrial function, also increased in this study following EPO treatment, highlighting potential benefits of EPO longer-term on the mitochondrial changes seen in FRDA. Unfortunately, due to the inconsistency between trials, with no large-scale trials or longer-term studies, EPO is not currently used clinically for FRDA. Many of the aforementioned trials were on a short-term basis, and it is thought that these changes in frataxin are only transient, if there is no change in symptoms, then the risks of potentially developing polycythaemia, which can lead to thrombosis or cardiovascular complications, may outweigh the potential benefits of EPO.

### Other clinical trials

Exogenous interferon γ 1b has been shown to increase frataxin messenger RNA and protein levels in FRDA patients. *In vivo* treatment with IFN γ increased frataxin expression in DRG neurons and improved the sensorimotor performance in FRDA mice.^106^ There have been a few *in vivo* studies with limited positive outcomes. An open-label trial in 12 children found that it was well tolerated, and there was a small but significant change in frataxin levels, but these were very small and varied between tissue types.^107^ It is hard to generalize the findings from this trial due to the low numbers of participants which included no placebo or comparison group. A larger double-blind, multicentre study, which was placebo-controlled, found no significant difference between groups in mFARS or frataxin levels in buccal or whole blood cells after 6 months of treatment.^62^

An alternative avenue is focusing on the association of FRDA with a high diabetes prevalence, therefore using medications used in diabetes. A report looked at the impacts of glucagon-like peptide-1 analogues in *in vivo* and *in vitro* models in patients with FRDA.^108^ They found that there was improved glucose homeostasis in frataxin-deficient mice through enhanced insulin content and secretion in pancreatic β cells. Specifically, they found that exenatide induced frataxin and ISC-containing proteins in β cells and sensory neurons in the DRG. The frataxin-inducing effect of exenatide was confirmed in a pilot trial in Friedreich ataxia patients, showing modest frataxin induction in platelets over a 5-week treatment course.

Further trials and a greater understanding of the causal factors contributing to the increased risk of diabetes mellitus in FRDA may enhance these trials and outcomes for therapeutic options.

Another medication that has been investigated is leriglitazone It is a selective peroxisome proliferator-activated receptor γ (PPARγ) agonist that is known to cross the blood–brain barrier, which has previously been a problem when developing medication for FRDA. It was investigated in a phase 2, double-blind, randomized controlled trial (FRAMES) for its potential benefits in FRDA.^109^ Preclinical models suggested that it could improve mitochondrial function by significantly increasing markers of mitochondrial biogenesis in FRDA patient cells.^110^ The study enrolled patients aged 12–60 years with a SARA score <25. The focus was on the change in spinal cord area at the C2-C3 level, of which they found no significant difference between the two groups at 48 weeks. However, iron accumulation was significantly higher in the placebo group in the dentate nucleus. Despite finding some positive changes in the phenotype when using leriglitazone, the sample size of 39 participants limits the statistical power of this report. Also, FRDA is a slowly progressive disease, and it would be useful to follow-up for a longer period than 48 weeks and assess how these features translate to clinical changes in symptoms for the patients, for example by using scales such as mFARS.

Linoleic acid was recently trialled in FRDA patients. Linoleic acid is an essential 18-carbon n-6 polyunsaturated fatty acid (PUFAs), and an adequate dietary supply of LA is crucial for human health.^111^ Linoleic acid has been focused on due to it being a PUFAs, which are essential for healthy neuronal, cardiac and skeletal muscle membranes, they are also particularly vulnerable to oxidative stress, due to their degree of unsaturation.^112,113^ The hypothesis therefore was that prevention of PUFA oxidation may aid with disorders caused by an imbalance in the redox state, such as FRDA. A specific synthetic, deuterated homologue of linoleic acid RT001 was recently trialled in a double-blind trial for FRDA (NCT04102501).^112^ It has previously been shown in various reports to reduce the production of lipid peroxidation in general yeast models of oxidative stress and multiple animal models.^113-121^ It has also been shown *in vitro* that RT001 specifically, reduces the oxidative stress associated with increased iron, therefore being an logical candidate for FRDA treatment.^118,119^ 45 FRDA patients completed the trial, aged 12–50 years, with symptom onset before 26 years of age.^112^ There was no significant benefit found with RT001 in this patient population, in fact mFARS values significantly improved in the placebo group in comparison to the treated patients. This may be due to the mismatching ages of the placebo group in comparison to the treated group, making it more difficult to draw direct comparisons, due to the results not allowing for different ages of onset, allele lengths and age at the time of the trial. Linoleic acid had previously been trialled in 22 patients with FRDA in 1982, there was also no significance found in performance scores in this smaller cohort, although this may be due to the lack of objective clinical tests and the form of linoleic acid used.^122^ This highlights the need for further progression in our understanding of the phenotype, to allow for a biological marker in order to assess whether the treatment efficacy.

There have been other clinical trials that, despite being safe and well tolerated, have been unable to demonstrate efficacy as treatments for FRDA. These include clinical trials of luvadaxistat,^123^ 5-hydroxytryptophan^124,125^ and lecithin.^126^ This is likely due to complexity of the phenotypical changes in FRDA, the lack of complete understanding of these specific changes, the difficulty in getting medications to target these specific changes in differing pathways without having off-target effects and causing side effects, and finally changes in these pathways not leading to translational clinical improvement in patients.

### Genetic or epigenetic therapeutic approaches

Another therapeutic approach relates to genetic or epigenetic therapy. Gene therapy has become more popular in recent years in neurodegenerative disorders, with risdiplam being successfully approved for spinal muscular atrophy, and more recently, tofersen gaining approval for SOD1-linked ALS, highlighting the growing impact of targeted genetic therapies in neurological diseases.^127^ As previously mentioned, FRDA is caused by a large expansion in GAA in intron 1 of *FXN*, leading to a reduction in frataxin expression. Therefore, gene therapy for FRDA could potentially be used to increase the frataxin expression at gene level.^128^ In theory, sustained restoration of frataxin, if done to a sufficient degree and at an early enough time point in the disease course and in sufficient numbers of cells, should provide an ideal treatment depending on adverse events. The common problems with gene therapy include being able to target the relevant or affected tissues, the potential to trigger a widespread and detrimental immune response, lack of long-term efficacy without adverse effects and the safety considerations. The complexity of the disease and potential off-target effects of gene-editing tools need to be assessed in detail.

The process involves using viral vectors, such as adeno-associated viruses (AAVs) to deliver a functional copy of the *FXN* gene and restore frataxin production. Preclinical studies have demonstrated that AAV vectors can successfully increase frataxin levels and mortality in animal models of FRDA.^129,130^ However, challenges include ensuring efficient and widespread delivery to affected tissues (such as neurons and cardiac muscle) and avoiding immune responses to the viral vector. Few AAV vectors readily target the brain without intraparenchymal administration; in mouse models, there has been some relative success in restoring cardiac function, and in FRDA mouse models,^129,130^ there have also been issues with development of immunity to AAVs associated with initial treatment, likely preventing repeat administration.^131^

One of the common problems with gene therapy is the potential for high levels of overexpression of frataxin causing further mitochondrial and cardiac toxicity.^132,133^ Belbellaa *et al*. investigated whether the use of *in vivo* AAV-mediated vectors for FRDA could be detrimental to frataxin-deficient *Mck* mice when compared to wild-type.^132^ They found that overexpression of frataxin below 9-fold was safe in mice, but beyond 20-fold it was toxic for the mitochondria and the heart, resulting in severe impairment of complexes I and II enzymatic activity, alteration of mitochondria ultrastructure, cardiomyocyte cell death and fibrosis. The mitochondria were seen to be swollen, with sparse cristae (correct cristae structure is vital for mitochondrial function) and the total volume occupied by the mitochondria in the cardiomyocytes appeared significantly increased. Despite clear conclusions and outcomes of dose-dependent reversal of FRDA cardiomyopathy symptoms, before toxicity levels, this study did not investigate the potential cytotoxic cell response targeting the transduced cardiomyocytes, or the innate or adaptive immune response against the vector. The study showed that the dose given is very important and this needs to be perfected before translation to human models. A further study also using the AVVrh10 vector in Mck-Cre-*FXN*(L3/L-) mice found that it fully prevented the onset of cardiac disease and in late administration, once the onset of heart failure had begun, it completely reversed the cardiomyopathy of these mice within a few days.^134^ The AVVrh10 is readily transported within the myocardium after IV injection, making it a viable option to prevent or treat cardiomyopathy in FRDA. They found robust expression of *FXN* in the liver and heart with substantial overexpression (more than tenfold), but lower expression in the skeletal system and the CNS. Further studies need to be done to increase the efficiency of the vector’s effects or expression in sensory neurons, to impact the ataxia phenotype. Despite lower expression in the skeletal and nervous systems, this report found the DRG were highly positive for GFP expression all along the vertebral column and axonal projections. The cardiac *Mck* conditional knockout mouse model used in this study is a good model for most of the features of FRDA cardiomyopathy; however, these models do not specifically manifest the sensory ataxia-like symptoms seen in FRDA. Piguet *et al*.^133^ developed a mouse model to express these ganglionopathic and sensory neuropathic changes that are associated with *FXN* deficiency. The *Pvalb-Cre* conditional knockout mice present with a more rapid and severe sensory ataxia, but with a similar clinical phenotype, to allow for the study of these mice. They found that a post-symptomatic single intravenous injection of AAV9-*CAG*-*FXN*-HN at a dose of 5 × 10^13^ vector genomes (vg)/kg led to a significant improvement of coordination in treated versus untreated mice in all tests performed. Interestingly, they found that mice injected at 3.5-week-old (early symptomatic mice) improved at 1 week of treatment, but by 8.5 weeks of age began to deteriorate again, progressively worsening, but not to the levels of the untreated mice. When injected initially at 7.5 weeks of age (late symptomatic), they were found to be completely corrected at 1 week of age and sustained this until time of euthanasia at ∼18.5 weeks of age. This mouse model demonstrated cerebellar ataxia with loss of *FXN* in Purkinje cells and deep cerebellar nuclei, and should be studied further in the future, to allow for evaluation of treatment effects on the cardiac and neurological effects associated with the disease.

Recent advances in gene therapy for FRDA have demonstrated the potential to correct mitochondrial cardiomyopathy through targeted interventions. A key study has shown that correcting half of the cardiomyocytes is sufficient to fully rescue FRDA-associated cardiac dysfunction via cell autonomous mechanisms.^135^ Researchers have defined the minimum vector biodistribution required to achieve therapeutic effects at different disease stages, highlighting the efficacy of the AAVrh10.CAG-hFXN-HA vector in the FRDA Mck model. Cardiac function rescue was observed in a dose-dependent manner, with a single intravenous injection of 5.4 × 10^13^ vg/kg at 7 weeks of age, completely reversing LV dilation and hypertrophy. Lower doses, such as 5 × 10^12^ vg/kg, still produced therapeutic effects, and four out of five mice receiving 1 × 10^13^ vg/kg survived up to 15 weeks, increasing lifespan by 57% despite severe cardiac dysfunction. Vector distribution in the heart was strongly correlated with transgene expression and the percentage of cardiomyocytes transduced. Importantly, correction of 50% of cardiomyocytes was sufficient for cardiac function restoration, regardless of neurological disease progression. The therapeutic outcome was primarily dependent on vector biodistribution within the heart rather than the absolute level of frataxin re-expression.^135^ Notably, the therapeutic threshold remained consistent regardless of the severity of cardiac dysfunction at the time of treatment, reinforcing the potential of gene therapy as a viable intervention for FRDA-related cardiomyopathy.

The current ongoing clinical trials looking at gene therapy specifically for cardiomyopathy in FRDA are NCT05302271 and NCT05445323.^136,137^ The first phase 1A and 1B study is using AAV rh10 vector. This study is investigating 25 participants, 18–50 years old and focusing on a dose escalation study design, to allow the identification of the lowest effective therapeutic dose. It will focus on the safety and follow patients up for 5 years. Similarly, NCT05445323 is investigating LX2006, in a dose-ascending, multicentre trial for safety and efficacy. This will also extend over a 5-year period. These clinical trials are vital to assess efficacy and safety and determine whether the gene therapy is of translational benefit. They are both due to be completed in 2029. As mentioned previously, gene therapy vectors are easily targeted towards the heart muscles, but effective targeting of neurons is more difficult. Therefore, current gene therapy approaches may benefit the cardiomyopathy and prolong life in FRDA but may not improve ataxia symptoms if unable to target DRG neurons and other affected CNS sites.

There is promise in developing a potential gene therapy, with progress towards developing a therapy targeting the cardiomyopathy specifically. Further optimization is needed to allow for an effective dose, without too many adverse effects or frataxin toxicity, the finalization of the timing of the treatment and how to target the exact location of the neurodegeneration pathology, not solely the heart.

An alternative method to avoid potential frataxin toxicity is using the CRISPR-Cas9 method, which can remove the expanded GAA repeats to restore normal gene function. Ouellet *et al*.^138^ focused on using this to delete the repeat expansions in the *FXN* gene, with the aim to restore normal *FXN* expression. The YG8sR mouse model was used as it exhibits more severe FRDA symptoms than other mouse models, including significant motor and coordination issues, glucose intolerance and greatly reduced expression of *FXN*. This study highlights a proof of concept of an approach for epigenetic therapy to avoid toxicity and potentially remove the GAA repeat. However, research needs to be done over a longer time period and a safe and efficient delivery method devised before such an approach could be trialled in humans.

Other forms of epigenetic therapy for FRDA have been researched by targeting HDAC inhibition to reactivate *FXN*. It is considered that gene silencing of *FXN* may occur through heterochromatin formation.^32,33^ Soragni *et al*. looked at 2-aminobenzamide-based HDAC inhibitors (HDACi) in patient-derived iPSCs to determine their effect on *FXN* gene expression and chromatin modifications.^34^ They found that the HDACi 109/RG2833 increased *FXN* mRNA levels and frataxin protein levels in the neuronal cell model. These positive changes were also seen in FRDA patients with an increase in *FXN* mRNA and H3 lysine 9 acetylation in PBMCs. This highlights the potential to modify chromatin structure and partially reverse *FXN* gene silencing. This study is a phase 1 trial for safety and proof of concept; larger further studies with longer term follow-up would be needed to optimize efficiency and assess longer term benefits.^34^ However, this provides alternative and positive avenues for potential epigenetic therapy in the future.

## Conclusion

Despite significant advances in our understanding of the pathophysiological changes in FRDA, many aspects of its complex phenotype remain poorly understood. The genetically inherited GAA-triplet repeats result in *FXN* deficiency, leading to several downstream effects, including decreased Nrf2 activity, iron accumulation and an imbalance in reactive oxygen species, all contributing to mitochondrial dysfunction. Gaining a deeper understanding of the specific pathophysiological processes at the mitochondrial and cellular levels will be crucial for advancing precision medicine approaches in FRDA, as seen in other neurological disorders such as Parkinson’s disease.

Current evidence suggests that Nrf2 activators hold promise as a therapeutic strategy for ameliorating oxidative stress in FRDA. While preclinical models have consistently demonstrated neuroprotective effects, clinical data remain limited and variable. The literature highlights both the therapeutic potential and the urgent need for more rigorous, longitudinal trials, particularly in paediatric populations, where studies are still ongoing, and recruitment is often challenging due to the rarity of the disease. This emphasizes the importance of multicentre collaboration design despite the logistical and methodological hurdles entailed. Research into frataxin replacement therapies also holds considerable promise for future treatment approaches. Future studies should focus on optimizing dosing regimens, ensuring long-term safety and addressing age-specific outcomes. Drawing insights from research in neurodegenerative diseases such as Alzheimer’s disease and amyotrophic lateral sclerosis could help further unravel the pathophysiological mechanisms and ultimately pave the way for the development of precision medicine treatments for FRDA.

## Supplementary Material

fcag143_Supplementary_Data

## Data Availability

The data that support the findings can be found in the references listed at the end of the review.
