# Supplementary material for: Recent developments in Friedreich’s ataxia: a state-of-the-art review
Source: Brain Commun. 2026 Apr 20;8(3):fcag143. doi: 10.1093/braincomms/fcag143 (PMC13180647; doi:10.1093/braincomms/fcag143)
Supplement: fcag143_Supplementary_Data [file fcag143_supplementary_data.pdf]

SUPPLEMENTARY TABLE 1 – FRDA MEDICATIONS TO DATE

Medications that have been trialled, their mechanism of action, the stage of clinical trial, the outcomes and the clinical trial numbers are listed in supplementary table 2.

Medication	Mechanism of action	Clinical trial phase	Outcomes	Adverse events
**Approved or used clinically**				
**Omaveloxolone**	Nrf2 activator	Phase 3<br><br>FDA approved in ≥16 years	• Significant change in mFARS at week 48<br>• Limited adverse events	Headache, fatigue, increase in liver transaminases (transient and reversible), and mild gastrointestinal (GI) symptoms.
**Coenzyme Q_{10}**	Antioxidant	Phase 3, used widely in clinical practice.	• No consistent neurological benefits after 6 months<br>• Mixed outcomes, not consistent.	Indistinguishable from placebo.
**Ongoing trials**				
**Vatiquinone**	Targets 15-lipoxygenase-regulator of ferroptosis	Phase 3 – granted fast track designation by US FDA.	• 24 months significant improvement in neurological function and disease progressive relative to natural history cohort.[1]	Well tolerated, mild GI symptoms.
**LX2006 (AAVrh.10hFXN)**	Gene therapy – AAV associated	Approved for Phase 2 accelerated approval pathway	• Mean reduction in left ventricular mass index over 6 months<br>• Increased post-treatment frataxin expression above baseline on myocardial biopsies.	Normal risks with AAV-related vectors, with the immune-suppression used.
**Nomlabofusp (CTI-1601)**	Protein replacement for FXN deficiency	Phase 2	• No reported adverse effects, in non-human primates had caused death<br>• Increase in frataxin protein levels from baseline vs placebo at day 14 initially.	Mild injection site reactions, mild GI or fatigue.
**DT-216**	GeneTAC small molecule designed to specifically target the GAA pathogenic repeat expansion	Phase 1/2	• No serious treatment-related adverse events.<br>• Significant 30% mean increase in *FXN* mRNA levels 2 days after third weekly dose vs placebo, also increased at 7 days.	Awaiting safety data.
**Calcitriol**	Active Vitamin D	Phase 4	• Statistically significant increase in frataxin levels after 12 months	Hypercalcaemia, GI symptoms.
**Etravirine**	NNRTI	Phase 2	• Increased frataxin protein both in vitro and in vivo.<br>• Significant change in progression of the SARA score, when comparing pre- and post-treatment.	Rash, GI symptoms, fatigue.
**Completed or withdrawn**				
**Leriglitazone**	Peroxisome proliferator-activated receptor γ (PPARγ) agonist	Phase 2	• No significant difference of change in spinal cord area at C2-C3 level<br>• Less accumulation of iron in the dentate nucleus compared to placebo group, not significant.	Weight gain, oedema, headache. Fluid retention monitored.

**Linoleic acid/RT001**	Polyunsaturated fatty acid	Phase 3	• No significance in change in maximum oxygen consumption rate from baseline at 11 months FA vs placebo<br>• No significant change from baseline in distance walked in 1 minute at 11 months	Mild GI upset, fatigue.
**Resveratrol**	Antioxidant	Phase 2	• No benefit to placebo.	GI upset
**Deferiprone**	Iron chelator	Phase 2	• 20mg/kg/day of deferiprone had a benefit in patients with less severe disease.[2] It led to an improvement in ICARS, mFARS and kinetic function – not with all doses.<br>• 20mg/kg/day or 40mg/kg/day dose both reduced the LV mass index, however the 40mg/kg/day dose appeared to worsen the mFARS and ICARS score. [2]	Neutropenia, agranulocytosis (rare), GI upset. Need haematological monitoring.
**INF-y 1b (Actimmune)**	Interferon	Phase 3	• Higher rate of adverse events.<br>• No significant change in mFARS from baseline at week 26, but a significant change at 12 weeks.<br>• Significant change in whole blood frataxin levels at 12 weeks from baseline.	Flu-like symptoms, fever, fatigue.
**Nicotinamide**	Vitamin B_3 complex	Phase 3	• Daily dosing at 3·5-6 g resulted in a sustained and significant upregulation of frataxin expression<br>• Reduction in heterochromatin modifications at the *FXN* locus<br>• No significant clinical change	GI symptoms, flushing, fatigue.
**(+) Epicatechin**	Anti-inflammatory, inhibits NF-kB signalling	Phase 2	• No significant change in mFARS from baseline or in ventricular hypertrophy.	Minimal reported.
**Methylprednis-olone**	Corticosteroid	Early Phase 1	• At 26 weeks compared to baseline timed 25-foot walk and FARS did not change significantly.<br>• At 26 weeks significant change in 1-minute walk in the paediatric cohort, otherwise no changes.[3]	Mood changes, hyperglycaemia, insomnia, weight gain.
**Carbamylated Erythropoietin/ Lu AA24493**	Anti-inflammatory	Phase 2	• Did not have a significant effect on *FXN*/functional rating scales	Injection related, headache, fatigue.
**Idebenone**	Antioxidant, short-chain analogue of CoQ10	Withdrawn	• No significant change in mFARS, ICARS or activities of daily living.	GI symptoms, headache.

References

Please see supplementary table 2 for the trial numbers, of which the table is a summary.

1. Zesiewicz T, Salemi JL, Perlman S, et al. Double-blind, randomized and controlled trial of EPI-743 in Friedreich's ataxia. *Neurodegener Dis Manag*. 2018;8:233-242. doi:10.2217/nmt-2018-0013
2. Pandolfo M, Arpa J, Delatycki MB, et al. Deferiprone in Friedreich ataxia: A 6-month randomized controlled trial. *Ann Neurol*. 2014;76:509-521. doi:10.1002/ana.24248
3. Patel M, Schadt K, McCormick A, Isaacs C, Dong YN, Lynch DR. Open-label pilot study of oral methylprednisolone for the treatment of patients with friedreich ataxia. *Muscle Nerve*. 2019;60:571-575. doi:10.1002/mus.26610

SUPPLEMENTARY TABLE 2 – CLINICAL TRIAL NUMBERS

Documents the clinical trial numbers for the ongoing or completed trials described in supplementary table 1.

**Medication**	**Clinical trial no**
**Omaveloxolone**	• NCT06054893 – Paediatric Clinical Trial (CHOP) (2-15 years)<br>• NCT02255435 – MOXIe (≥ 16 years)<br>• NCT06623890 – recruiting for long-term safety (≥16 years)
**Coenzyme Q_{10}**	ISRCTN87024790 (≥ 10 years)
**Idebenone**	• NCT00905268 – Efficacy and safety (≥ 8 years)<br>• NCT00078481(≥ 5 years)<br>• NCT00229632 (9-18 years)<br>• NCT00537680 (8-17 years)<br>• NCT00697073 – safety and tolerability (8-18 years)<br>• NCT00015808 (≥ 5 years)<br>• NCT00993967 – long-term safety and tolerability (≥ 9 years)
**Calcitriol**	• NCT04801303 (16-65 years)
**Resveratrol**	• NCT05285540 (18-55 years)<br>• NCT01339884 (≥ 18 years)<br>• NCT03933163 – micronized (≥ 16 years)
**DT-216**	• NCT05285540 (18-55 years)<br>• NCT06874010 – not yet recruiting, safety, tolerability (18-55 years)
**Deferiprone**	• NCT00530127 – safety and tolerability (7-35 years)<br>• NCT00897221 – safety and efficacy (7-35 years)
**Carbamylated Erythropoietin/Lu AA24493**	NCT01016366 (≥ 18 years)
**INF-γ 1b/ACTIMMUNE**	• NCT02797080 – long-term safety extension for children and young adults (11-27 years)<br>• NCT01965327 (5-17 years)<br>• NCT02035020 – safety and efficacy (18-45 years)<br>• NCT03888664 – open trial (12-50 years)<br>• NCT02415127 (10-25 years) and NCT02593773 (10-26 years) – safety, tolerability and efficacy (STEADFAST)
**Etravirine**	NCT04273165 (10-40 years)
**Lerglitazone**	FRAMES (12-60 years)[108]
**Linoleic acid/RT001**	• NCT02445794 – Phase 2 (18-50 years)<br>• NCT04102501 – safety and tolerability – Phase 3 (12-50 years)
**Methylprednis-olone**	NCT02424435 (≥ 5 years)
**Nicotinamide**	• NCT04817111 (18-64 years)<br>• NCT01589809 (≥ 18 years)<br>• NCT03761511 – withdrawn due to funding (≥ 18 years)
**(+) Epicatechin**	NCT02660112 (10-50 years)
**Vatiquninone**	NCT04577352 – MOVE-FA (≥ 7 years)
**LX2006 (AAVrh.10hFXN)**	• NCT05445323 – still active (18-50 years)<br>• NCT05302271 – recruiting (18-50 years)
**Nomlabofusp (CTI-1601)**	• NCT06447025 – enrolling (≥ 18 years)<br>• NCT06681766 – children, recruiting (2-17 years)<br>• NCT05579691 (≥ 18 years)<br>• NCT04176991 (≥ 18 years)<br>• NCT04519567 – multiple ascending doses (≥ 18 years)
